# Effect of Nonionic Surfactants (Dodecyl Maltoside and Polysorbate 20) on Prevention of Aggregation and Conformational Changes of Recombinant Human IFNβ_1b Induced by Light

**Published:** 2017

**Authors:** Najmeh Mahjoubi, Ahmad Fazeli, Rassoul Dinarvand, Mohammad Reza Khoshayand, Maryam Shekarchi, Mohammad Reza Fazeli

**Affiliations:** a *Department of Drug and Food Control, Pharmaceutical Sciences Research Center, Faculty of Pharmacy, Tehran University of Medical Sciences, Tehran, Iran. *; b *Research and Development Department, Zistdaru Danesh Pharmaceutical Company. Tehran, Iran.*; c *Department of Pharmaceutics, Faculty of Pharmacy, Tehran University of Medical Sciences, Tehran, Iran.*; d *Food and Drug Research Center, Food and Drug Organization, MOH&ME, Tehran, Iran. *; e *Department of Drug and Food Control, Pharmaceutical Quality Assurance Research Center, Faculty of Pharmacy, Tehran University of Medical Sciences, Tehran, Iran.*

**Keywords:** Nonionic surfactant, Circular dichroism (CD), oxidation, anti-aggregation, rhIFN β_1b

## Abstract

Liquid protein formulations are prone to form aggregates. The effect of nonionic surfactants such as Polysorbate 20 (PS 20) and n-Dodecyl β-D-maltoside (DDM) on the prevention of aggregation and conformational changes of recombinant human IFNβ-1b (rhIFN β_1b) was explored. Polysorbate has been used in formulations of protein pharmaceuticals. There have been concerns about using PS 20 due to its residual peroxide content which may negatively affect protein efficacy. n-Dodecyl β-D-maltoside has been of interest and shown to be highly effective in prevention of aggregation. Fresh bulk of rhIFN β_1b was formulated using DDM or different concentrations of PS 20. Formulations were exposed to light stress condition according to the ICH guideline of Q1b. The overall conformational integrity of individual samples was characterized by a combination of Circular dichroism (CD), Fluorescence spectroscopy and RP_HPLC techniques.

The CD spectrum depicting the conformational integrity of rhIFN β_1b showed 31.9% and 31.2% decreases in α-helix content of protein samples with 0.2% or 0.02% of PS20 compared to only18.2% of that containing 0.2% DDM. The RP-HPLC analysis also showed that the oxidized impurity in formulation containing DDM is less than those contain PS 20.

Complementary analysis of the liquid formulations using IFR and UV methods also was in compliance with the data obtained by CD.

Compared to PS 20, the sample of rhIFN β_1b formulation with DDM was more resistant to the destruction effect of light. Results were in accordance with previous studies and could suggest DDM as a reliable anti-aggregation surfactant in biopharmaceutical formulations.

## Introduction

Nonionic surfactants of PS 20 and polysorbate 80 (PS 80) are widely used in protein formulations to prevent aggregation because of their effectiveness at low concentrations, low toxicity and their ability to minimize protein surface adsorption and aggregation under various processing condition (1-3). Most of the marketed monoclonal antibodies formulations contain one of these surfactants (4). PS 20 is used in some cytokines such as Actimmune1 (IFN–gamma-1b) and Avonex (IFN β_1a) as well as Ontak1 (Denileukin diftitox) (5, 6).

Several mechanisms for protein stabilization by surfactants have been proposed. Nonionic surfactants confer surface-induced anti-aggregation behavior by competing with proteins for interfaces which could initiate aggregation (7). Binding to hydrophobic regions on the protein surface and reduction in intermolecular interactions (8-10), increase in the free energy of protein unfolding (11) and acting as chemical chaperone (12, 13) have also been reported as possible anti-aggregation mechanisms of surfactants. They can also modulate adsorption loss and aggregation by coating interfaces and/or participating in protein–surfactant associations (14). Inherent impurities within the surfactants such as PS 80 can also influence significantly photostability of a protein (15). Commercial polysorbates contain a large amounts of polyoxyethylene, sorbitan polyoxyethylene, and isosorbide polyoxyethylene fatty acid esters (16, 17, 1). These products may contain residual amounts of peroxides that in aqueous solution could react and damage proteins (18-20).

 Therapeutic proteins are prone to be oxidized during different manufacturing steps and storage, due to exposure to intense light, trace amounts of metal ions or peroxide impurities in polysorbate excipients (21-23). Amino acids containing either sulfur atom (cysteine and methionine) or an aromatic ring (histidine, tryptophan, tyrosine and phenyl alanine) are at most risk of oxidation (24). For the analysis of protein circular dichroism, it is observed amino acids with aromatic functionality in protein such as tryptophan (Trp) is likely to make significant contribution. With a light source at an emission spectrum of greater than approximately 300 nm, Trp is the major amino acid residue in proteins with significant absorbance (25, 26). More often oxidation increase the propensity to aggregates. Oxidation of Trp residues and subsequent aggregation of the model therapeutic protein type I soluble tumor necrosis factor receptor has been reported to be induced by UV light at 302 nm (5). Peroxodisulfate-mediated oxidation of recombinant human interleukin-2 altered secondary structure of the protein, as observed by far-UV CD (27). 

Potential problems with using polysorbates and other polyoxyethylene-containing surfactants make clear the need for alternative surfactants that prevent aggregation and unintended protein damage. To circumvent the effect of residual inherent oxidative species within the surfactants, one of alternatives is a class of non–ether-based alkylsaccharide surfactants composed of a sugar moiety coupled to an alkyl chain that show significant improvement in stability and reduced immunogenicity (28). A broad range of alkylsaccharides (nonionic surfactants) comprise_Intravail alkylsaccharide excipients, have been studied as transmucosal absorption facilitator (29-31). It was observed that some of these molecules such as DDM have significant anti-aggregation effect (28). n-Dodecyl-β-D-maltoside is a non-toxic, non-mutagenic, and non-irritating and nonionic suger-based surfactant (28, 32) that finally metabolizes into CO2 and H2O through the corresponding sugar and fatty acid (28, 32). 

Although there are several liquid protein formulations in the market that have used PS 20 as an anti-aggregation agent (6, 33), incorporation of DDM in biopharmaceutical formulation has not yet received much attention due to scarce research.

However, one problematic issue in using polysorbates for biopharmaceutical is their potential adverse effect on protein stability, which has not been extensively reported. There are few examples of oxidate damage of recombinant human ciliary neurotrophic factor in solution and recombinant human granulocyte colony-stimulating factor in solution due to presence of residual peroxides in PS 80 (34). Specific products have recently been addressed, including monoclonal antibodies, calcitonin, granulocyte colony-stimulating factor, growth hormone, insulin, interferon alpha and beta, oxytocin and parathyroid hormone (35). The importance of different concentrations of PS 80 as a polyoxyethene-containing additive in formulation of Monoclonal antibody MAb1 has been investigated under stress condition (36).

Effect of non-ionic surfactants (DDM and PS 20) on prevention of aggregation and conformational changes in liquid formulation of recombinant human IFNβ_1b induced by light has been explored in current study. 

## Experimental

*Materials*

Bulk of rhIFN β _1b in solution at a concentration of 0.90 mg /mL was obtained as a gift from Zistdaru Danesh Co. Ltd. n-Dodecyl β maltoside, sucrose and glycine were purchased from Sigma-Aldrich (St. Louis, MO, USA). PS 20 was bought from Merck. Other reagents/chemicals were of analytical grade. 

*Sample preparation*

Three formulations of IFN β _1b at the protein concentration of 0.25 mg/mL were prepared containing 200 mM glycine and 200 mM sucrose. The individual formulations also had either 0.2% DDM, 0.2% PS 20 or 0.02% PS 20, while the pH was adjusted at 4.5. 

*Photostability*

Aliquots of 1 mL of the individual formulations were put in 2 mL type 1 glass vials before exposure to the light stress according to the ICH guideline of Q1b. Both the dark control vials were wrapped carefully with aluminum foil and unwrapped light-exposed vials were placed in an in-house cabin mimicking the ICH ES2000 bench-top photo-chamber equipped with both a cool white fluorescent bulb and a near ultraviolet (UV) lamp. Under the ICH guideline, formulations were exposed with 1.2 million lx h of cool white light and 200 Wh/m2 of near UV light at the temperature of 25 ^o^C. 

*Visual inspection*

Formulations were inspected visually against a dark background just after the light exposure.

*UV spectroscopy*

Ultra violet spectra of dark control and light-exposed of rhIFN β_1b fomulations with either 0.2% PS 20 or 0.02% PS 20 or 0.2% DDM at the wavelength range of 240–370 nm, in a 8-well quartz cuvette with a 1-cm path length were prepared using a Carry UV/VIS spectrophotometer (Varian, Australia). 

*Intrinsic Fluorescence (IFR)*

The tertiary structure of both light-exposed and dark control formulations was evaluated by setting the excitation wavelength at 285 nm and measuring the emission spectra collected from 300–400 nm using a Cary Eclipse (Varian, Australia) spectrofluorimeter equipped with a temperature controller bath. The relative fluorescence of the formulations at maximum emission was then calculated.

*Circular dichroism spectroscopy*

Circular dichroism technique was used to probe the secondary structural changes in protein conformation of both light-exposed and dark control formulations during experiments. Denaturation events were expressed as ellipticity parameter using a Jasco J-810 CD spectropolarimeter in the far-UV regions (190–260 nm). Changes of ellipticity at 222nm were selected to analyze the opening up of helical regions in the protein structure (37). The method of Bohm and others employing CDNN CD Spectra Deconvolution Software (available http://bioinformatik.biochemtech.unihalle. de/cdnn) was applied to quantify the structural changes(36).

*Reversed-Phase High Performance Liquid Chromatography (RP-HPLC)*

Reversed-Phase HPLC method was used to detect oxidized rhIFN β -1b in individual formulations. A Jupiter 300 (5 μM, 250*4.6 mm) C4 column was used in combination with a Security Guard (4*3 mm) C4 guard column (Phenomenex, Amstelveen, the Netherlands). The mobile phase was at a flow rate of 1 mL/min by a Waters 600 controller coupled to a Waters 717 Plus autosampler, and chromatograms were recorded with a UV detector (Waters 2487) at a wavelength of 214 nm. The eluents contained 10% acetonitrile with 0.1% trifluoroacetic acid (TFA) and 100% acetonitrile with 0.1% TFA. An elution gradient was applied according to Geigert at al. (27) 

## Results and Discussion

*Photostability study*

*Effect of Light Exposure on Turbidity*

**Figure 1:**
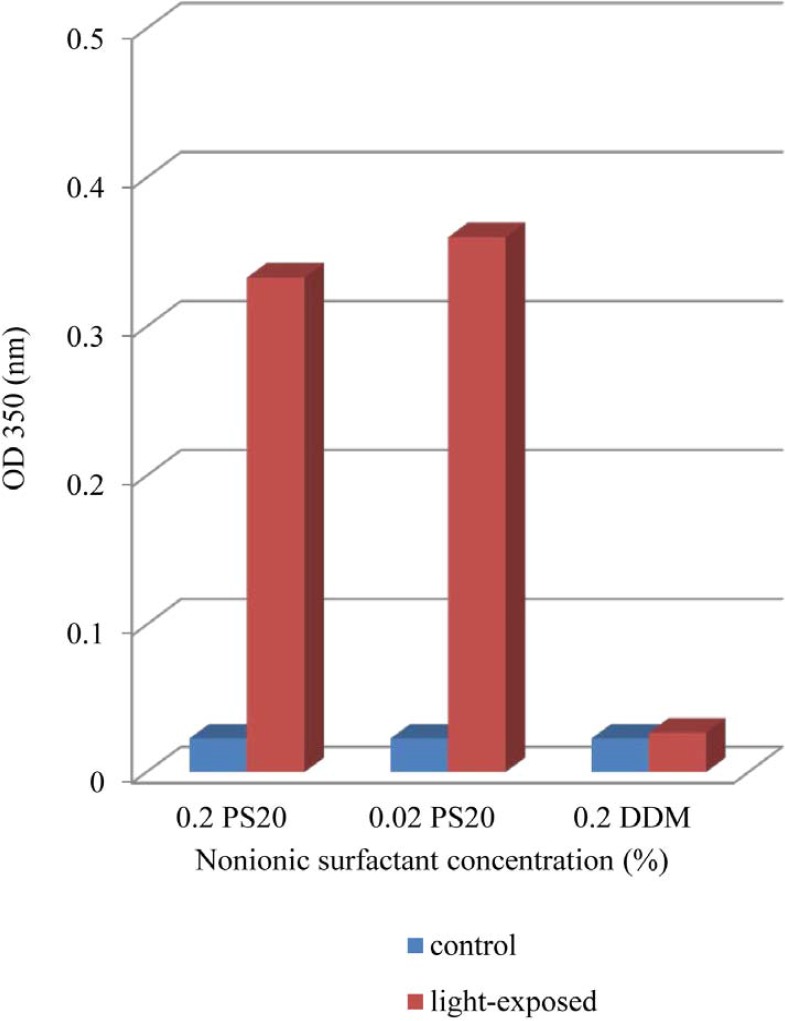
Turbidity for dark control and light-exposed formulations as a function of nonionic surfactant concentration (DDM or PS20).Turbidity was assessed by absorbance at 350nm (OD350). Formulations contained either 0.2% PS20 or 0.02% PS20 or 0.2% DDM.

**Figure 2:**
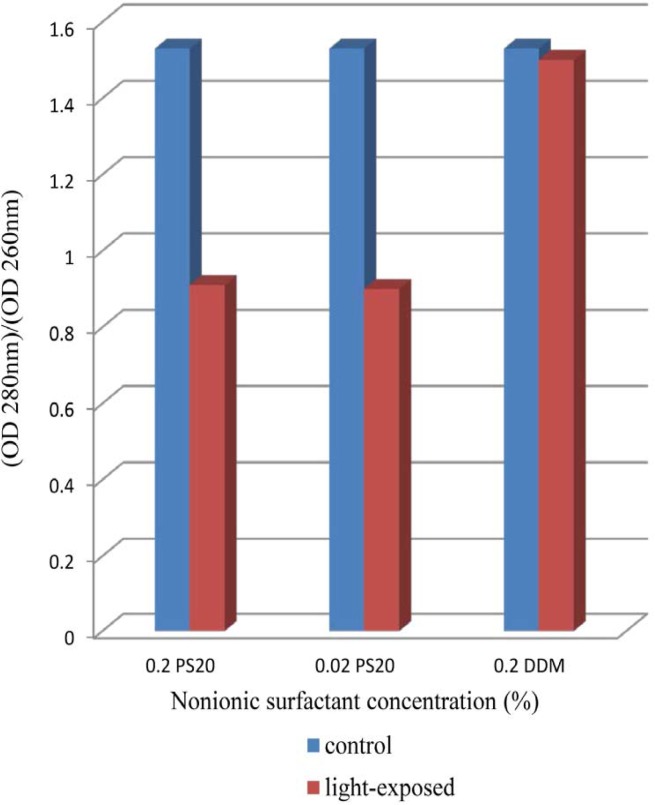
OD 280nm/OD 260nm for dark control and light-exposed formulations as a function of nonionic surfactant concentration (DDM or PS20). Formulations contained either 0.2% PS20 or 0.02% PS20 or 0.2% DDM.

The results indicate clearly an increase in OD 350 values in formulations containing both 0.02% and 0.2% of PS 20 while that containing 0.2% DDM remained almost at dark control level (Figure 1). In previous report, UV spectra showed broad absorbance peaks around the wavelength of 280 nm, indicate tryptophan, tyrosine or phenylalanine residues or disulfide bonds in the rhIFNβ-1b formulations (ure 2). Protein aggregation causes scattering of light that could lead to an increase in optical density (OD). Several factors, such as aggregate size, shape and protein concentration will affect the intensity of light scattering. Protein aggregates will lead to an increase in the optical density at 350nm (OD 350) while the ratio of 280/260 nm (OD 280/OD 260) decreases (38-41). Recombinant human IFN β-1b formulations contain PS 20 showed lower OD 280/OD 260, propably by absorption flattening due to extensive aggregation (Figure 2). These results tend to suggest that PS 20 has weak conservation effect on rhIFN β-1b compared to DDM and also this effect is fairly independent of the concentration used. 

*Effect of Light Exposure on* *tertiary* structure 

**Figure 3:**
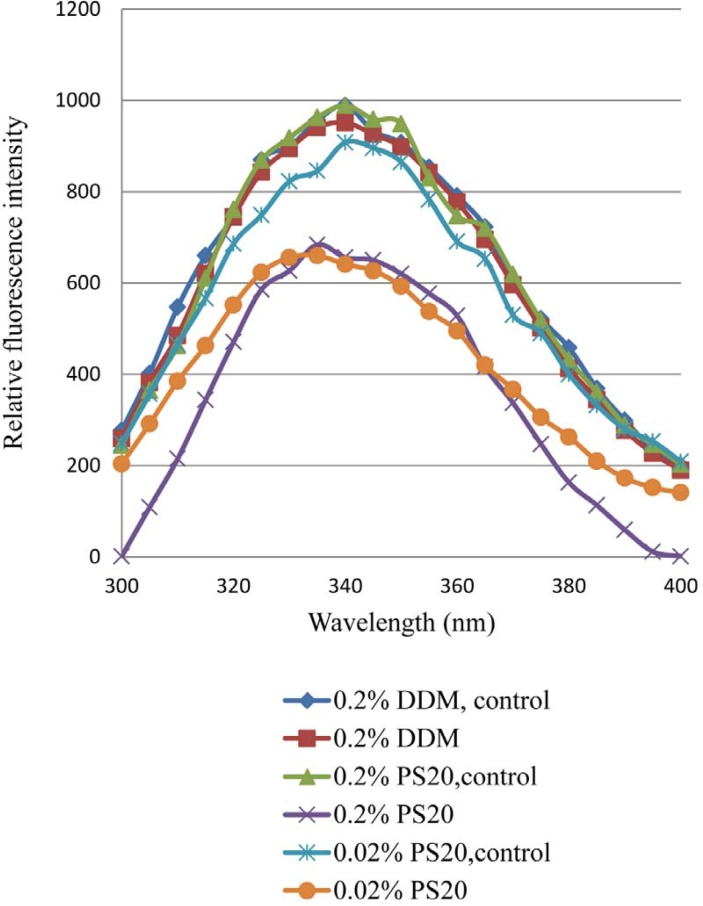
Fluorescence emission spectra of dark control and light-exposed formulations containing nonionic surfactant (DDM or PS20). Formulations contained either 0.2% PS20 or 0.02% PS20 or 0.2% DDM.

The effect of light exposure on the tertiary structure of rhIFN β-1b formulations with either 0.2% PS 20 or 0.02% PS 20 or 0.2% DDM has been shown in Figure 3. The tertiary structure of the protein was highly affected in formulations containing different concentrations of PS 20 compared to DDM. Following light exposure, the decreased fluorescence intensity with PS 20 was observed, which could be reflection of conformational changes and/or formation of aggregates due to oxidation of protein in the presence of PS 20. At a wavelength of 285 nm the tryptophans of rhIFNβ_1b formulations with PS 20 or DDM were excited, and a typical emission maximum around 340 nm was observed (Figure 3). This observation indicated a blue shift (towards to shorter wavelength) of the emission spectrum of both formulations containing PS 20, most likely due to an overall increased hydrophobic environment, and/or chemical degradation after light exposure. Fluorescence spectroscopy showed a loss of quenching Trp fluorescence after photooxidation (42-43). The wavelength of the fluorescence peak and its intensity provide a background of the Trp at position 22 (Trp22), which is close to the receptor binding site exposed to the solvent, and those of the tryptophans at positions 79 and 143, are inside the hydrophobic core of the protein which are stabilized through several hydrogen bonds and the disulfide bridge (44-46). Alterations in secondary and tertiary structure of human relaxin and increased exposure of its hydrophobic surface due to metal-catalyzed oxidation of lead was reported to promote aggregation and precipitation (47). 

**Figure 4:**
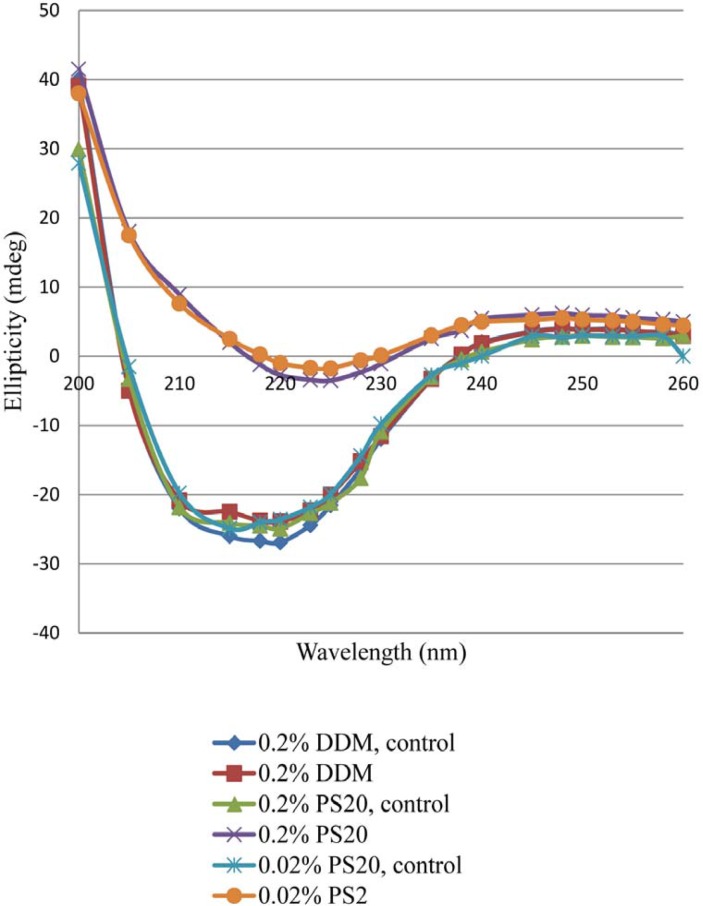
Far-UV CD spectra for dark control and light-exposed formulations containing nonionic surfactant (DDM or PS20). Formulations contained either 0.2% PS20 or 0.02% PS20 or 0.2% DDM.

**Figure 5:**
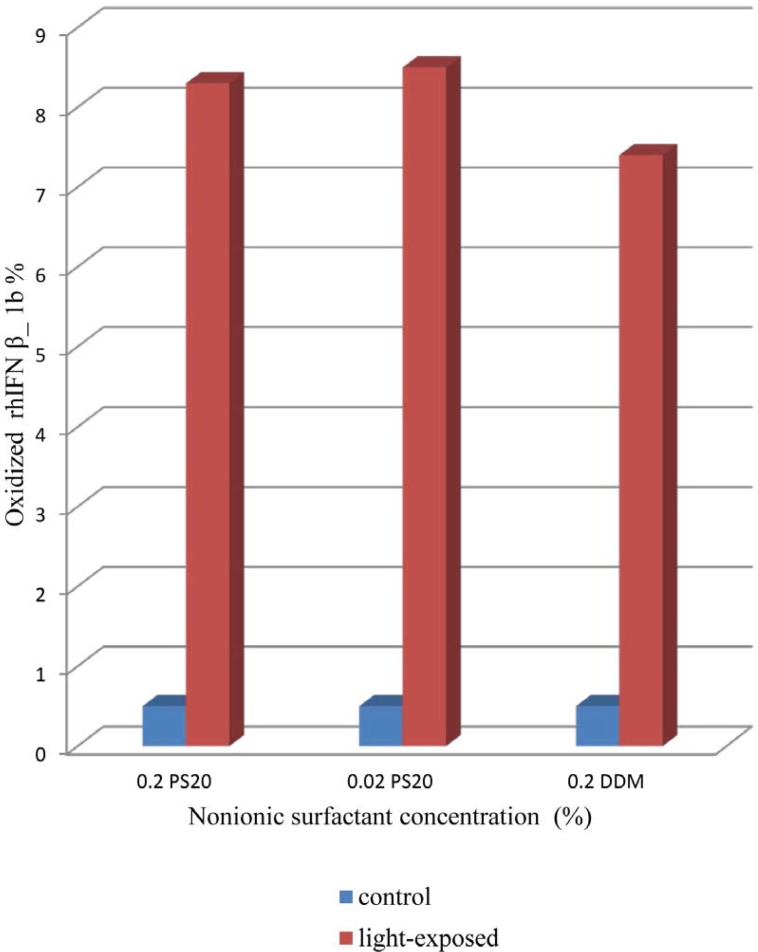
Oxidized rhIFN β_1b detected by RP-HPLC analysis. Formulations containing either 0.2% PS 20 or 0.02% PS20 or 0.2% DDM, respectively.

The fluorescence emission maximum peak of both rhIFNβ-1b formulations with 0.2% and 0.02% of PS 20 showed significantly decrease compared to formulation with 0.2% DDM, which may be attributed to higher degree of aggregation (48-49).

*Effect of Light Exposure on secondary Structure*

There were significant changes of ellipticity at 222 nm in the CD spectra of the light-exposed formulations. The intensity of far-UV CD spectra decreased significantly with rhIFN β_1b formulations containing 0.2% PS 20 or 0.02% PS 20 compared with 0.2% DDM (Figure 4). A negative shift in ellipticity at 222 nm wavelength represents more α-helix in protein secondary structure (50). The presence of 0.02% and 0.2% of PS 20 led to 31.9% and 31.2% decrease in α-helical structures of protein respectively, while only18.5% decrease in α-helical structure of protein was observed with DDM. The intensity of the spectrum decreased significantly in both rhIFNβ_1b formulations with 0.2% and 0.02% of PS 20 compared to 0.2% of DDM. This suggests that PS 20 has a fair protein stability when exposed to light (Figure 4). The CD analysis showed that the secondary structure of rhIFN β_1b in formulation containing DDM remained more intact compared to the one contains PS 20. This may be due to lower destabilizing effect of DDM after the light exposure.

*Reversed-Phase High Performance Liquid Chromatography*

In RP-HPLC analysis of rhIFNβ-1a, an oxidized protein has been reported to elute prior to the main peak, followed by several other peaks containing oligomerized potein (21). As shown in Figure 5. the content of oxidized rhIFN β_1b formulation with DDM is less than formulations containing 0.2% of PS 20 as well as 0.02% of PS 20. This event could be due to reports of the potentially oxidizing effect of PS 20 on protein due to the formation of peroxides on light exposure (5, 27).

The results obtained by UV, HPLC and IFR indicating that formulations containing PS 20 were more prone to conformational changes and aggregation on light induced interferon β_1b compared to DDM, also seen by CD analysis.

## Conclusion

The effect of different nonionic surfactants on stability and aggregation behavior of rhIFN β_1b was investigated under light stress condition. Less aggregation in the formulation containing 0.2% of DDM was observed compared to those harboring 0.02% and 0.2% of PS 20 determined by turbidity analysis of the samples by UV. The results obtained by HPLC and IFR indicate that formulations containing PS 20 were more prone to destabilization and aggregation compared to DDM, as also seen by CD spectra. Lower concentration of PS 20 in rhIFN β_1b formulation cannot be necessarily more effective than higher concentration of PS 20 compared to DDM as well.

Although PS 20 is mainly used in protein formulations, the findings in this manuscript demonstrate the importance of carefully choosing of surfactant in rhIFN β_1b formulation by light stress. However, it is still too early to conclude a possible substitution of PS 20 with DDM in protein liquid formulations but further studies may pave the way for its extensive usage in biopharmaceuticals. 
